# Targeting 3CLpro and SARS-CoV-2 RdRp by *Amphimedon* sp. Metabolites: A Computational Study

**DOI:** 10.3390/molecules26123775

**Published:** 2021-06-21

**Authors:** Nourhan Hisham Shady, Alaa M. Hayallah, Mamdouh F. A. Mohamed, Mohammed M. Ghoneim, Garri Chilingaryan, Mohammad M. Al-Sanea, Mostafa A. Fouad, Mohamed Salah Kamel, Usama Ramadan Abdelmohsen

**Affiliations:** 1 Department of Pharmacognosy, Faculty of Pharmacy, Deraya University, Universities Zone, New Minia City 61111, Egypt; Norhan.shady@deraya.edu.eg (N.H.S.); mskamel@yahoo.com (M.S.K.); 2 Department of Pharmaceutical Organic Chemistry, Faculty of Pharmacy, Assiut University, Assiut 71526, Egypt; alaa_hayalah@yahoo.com; 3 Pharmaceutical Chemistry Department, Faculty of Pharmacy, Sphinx University, New Assiut 71515, Egypt; 4 Department of Pharmaceutical Chemistry, Faculty of Pharmacy, Sohag University, Sohag 82524, Egypt; mamdouhfawzy3@yahoo.com; 5 Department of Pharmacy Practice, College of Pharmacy, AlMaarefa University, Ad Diriyah 13713, Saudi Arabia; mghoneim@mcst.edu.sa; 6 Department of Pharmacognosy, Faculty of Pharmacy, Al-Azhar University, Cairo 11371, Egypt; 7 Institute of Molecular Biology of NAS RA, Yerevan 0014, Armenia; garri.chilingaryan@rau.am; 8 Institute of Biomedicine and Pharmacy, Russian-Armenian University, Yerevan 0051, Armenia; 9 Department of Pharmaceutical Chemistry, College of Pharmacy, Jouf University, Aljouf 72341, Saudi Arabia; mmalsanea@ju.edu.sa; 10 Department of Pharmacognosy, Faculty of Pharmacy, Minia University, Minia 61519, Egypt; m_fouad2000@yahoo.com

**Keywords:** COVID-19, coronavirus, molecular docking, *Amphimedon* sp., sponge

## Abstract

Since December 2019, novel coronavirus disease 2019 (COVID-19) pandemic has caused tremendous economic loss and serious health problems worldwide. In this study, we investigated 14 natural compounds isolated from *Amphimedon* sp. via a molecular docking study, to examine their ability to act as anti-COVID-19 agents. Moreover, the pharmacokinetic properties of the most promising compounds were studied. The docking study showed that virtually screened compounds were effective against the new coronavirus via dual inhibition of SARS-CoV-2 RdRp and the 3CL main protease. In particular, nakinadine B (**1**), 20-hepacosenoic acid (**11**) and amphimedoside C (**12**) were the most promising compounds, as they demonstrated good interactions with the pockets of both enzymes. Based on the analysis of the molecular docking results, compounds **1** and **12** were selected for molecular dynamics simulation studies. Our results showed *Amphimedon* sp. to be a rich source for anti-COVID-19 metabolites.

## 1. Introduction

Coronavirus disease 2019 (COVID-19) is a severe and highly infectious viral disease that recently appeared in Wuhan, China [1,2,3]. Global health concerns have been raised by the COVID-19 pandemic [2], which has necessitated the search for drug candidates to help reduce its consequences. Certain natural products have been reported to act as potent drugs against COVID-19 [4,5,6,7,8,9]. Marine sponges are considered great reservoirs for potent antiviral chemicals [10,11,12,13]. Among them, *Amphimedon* sp. is a rich source of potential drugs, with diverse chemical constituents exhibiting a wide range of pharmacological activities [14,15]. We previously studied the antiviral potential of *Amphimedon* sp., and our results indicated that *Amphimedon* sp. total extract and petroleum ether fractions, as well as their derived nanoparticles, had anti-HCV potential, with pyrinodemin D, nakinadine B, and 3,4-dihydro-6-hydroxymanzamine A being the most potent anti-HCV agents [16]. This prompted us to study the ability of isolated compounds to act as inhibitors of COVID-19. Additionally, an in silico analysis using molecular docking studies was performed to provide mechanistic insight into the compounds’ potential as anti-COVID-19 agents. Owing to the high cost of laboratory and clinical trials, which are also time consuming and carry a high probability of false results, numerous bioinformatics methods have been used in the design of new drugs. Molecular docking is the most important bioinformatics tool used in drug design. Moreover, we studied the ADMET properties of our promising compounds to investigate the pharmacokinetic predictions for them. Compounds **1** and **12** were then selected for molecular dynamics simulation studies. It appears that the most efficient method for constructing an anti-2019-nCoV drug is to screen drugs which are currently being used in the clinic. RNA-dependent RNA polymerase (RdRp) is an essential protease that catalyzes the replication of RNA from an RNA template, and is therefore an attractive therapeutic target for tackling coronavirus. Hence, the anti-HCV effects of the fourteen isolated compounds encouraged us to further examine their ability to act as inhibitors of RNA-dependent RNA polymerase (RdRp) in COVID-19, via an in silico analysis using molecular docking studies, with the aim of ultimately finding an effective treatment for COVID-19 infections.

## 2. Material and Methods

### 2.1. Sponge Material

The marine sponge *Amphimedon* sp. was collected from Sharm El Shaikh (Egypt). It was then air-dried and stored at −24 °C until further investigation. Voucher specimens with registration numbers BMNH 2006.7.11.1 and SAA-66 were obtained from the Natural History Museum (London, UK) and the Pharmacognosy Department (Faculty of Pharmacy, Suez Canal University, Ismailia, Egypt), respectively.

### 2.2. Extraction and Isolation

The sponge material was freeze-dried (6 g) then extracted using methanol–methylene chloride. We fractionated the resulting crude extract between water and petroleum ether, yielding a petroleum ether fraction, then dichloromethane, ethyl acetate, and finally, butanol fractions. After concentration under a vacuum, petroleum ether (1 g), dichloromethane (250 mg), ethyl acetate (250 mg), and butanol (1 g) were produced. The petroleum ether fraction was chromatographed on a silica-gel column (gradient elution of petroleum ether: EtOAc, then EtOAc), followed by methanol, which was then chromatographed on sephadex LH-20 apparatus (Merck, Bremen, Germany). Final purification was performed on semi-preparative HPLC with acetonitrile (MeCN) and water as a mobile phase complemented by 0.05% trifluoroacetic acid with a gradient elution of 10% MeCN-H_2_O to 100% MeCN over 30 min at a flow rate of 5 mL/min, to yield compounds **1**–**14**.

### 2.3. Sequence Alignment and Modeling

Most of the promising clinical trials for the treatment of COVID-19 have highlighted important antivirus drug target proteins such as 3CLpro and RdRp, which are highly conserved between SARS-CoV and 2019-nCoV, particularly in terms of functional sites. Therefore, our docking study focused on these two important targets. Recent studies have highlighted that SARS-CoV-2 genes share nucleotide identity and display 89.10% nucleotide similarity with SARS-CoV genes [2,17]. The SARS-HCoV solved structure (PDB ID: 6NUS, chain A) was used for binding since it was the most sequelogous solved structure (97.08% sequence identity) to SARS-CoV-2 RdRp. Moreover, 6NUR is a SARS-HCoV non-structural protein 12 (nsp12) solved structure (cryo-electron microscopy) [3]. Remdesivir, an antagonist of 2019-nCOV RdRp, was chosen as a control. The model of the COVID-19 main protease was downloaded from the Protein Data Bank (www.rcsb.org accessed on 17 February 2021). The crystal structure of COVID-19’s main protease in complex with an inhibitor, N3 (PDB ID: 6LU7, chain A) [18] was chosen as the model for 3CLpro, and the inhibitor N3 was removed. The ligand N3 (*N*-[(5-methylisoxazol-3yl)carbonyl]alanyl-l-valyl-n~1~-((1*R*,2*Z*)-4-(benzyloxy)-4-oxo-1-{[(3*R*)-2-oxopyrrolidin-3-yl]methylbut2-enyl)-l-leucinamide) was used as a control.

### 2.4. Molecular Docking Methodology

Docking analysis was carried out using Discovery Studio 2.5 software (Accelrys Inc., San Diego, CA, USA) and a fully automated docking tool using the “Dock ligands (CDOCKER)” protocol, running on an Intel^®^ core^TM^ (Santa Clara, CA, USA) i32370 CPU @ 2.4 GHz 2.4 GHz, RAM Memory 2 GB, under the Windows 7.0 system. The docked compounds were built using Chem. 3D ultra 12.0 software (Chemical Structure Drawing Standard; Cambridge Soft Corporation, Cambridge, MA, USA (2010)), and copied into the Discovery Studio 2.5 software suite. An automatic protein preparation module was used when applying an MMFF94 forcefield. The binding site sphere was defined automatically by the software. Next, the receptor, having been prepared as described above, was entered as the input for the “input receptor molecule” parameter in the CDOCKER protocol parameter explorer. Force fields were applied to the test compounds to obtain the lowest-energy structure. The obtained poses were studied, and those showing the best ligand–HDAC interactions were selected and used for CDOCKER energy (protein–ligand interaction energy) calculations. The receptor–ligand interactions of the complexes were examined in 2D and 3D [19,20,21,22,23].

### 2.5. Prediction of Pharmacokinetic Properties and Toxicological Properties ADME/T

Online property prediction was used to calculate the pharmacokinetic properties of the isolated compounds under investigation. The following properties were studied: for absorption—water solubility, Caco-2 permeability, intestinal absorption (human), skin permeability and P-glycoprotein interactions; and for distribution—VDss, Fu, Log BB and CNS permeability. In addition, metabolism and excretion were studied, and pkCSM pharmacokinetics used to predict the toxicity of the compounds, including hepatotoxicity, skin sensitization, etc. The results were analyzed by comparing the values obtained for the investigated compounds to the reference values from the pkCSM pharmacokinetics online property prediction [24].

### 2.6. Molecular Dynamics Simulations

The docking binding poses of compound **2** for 6lu7 and compound **3** for 6nur were used as starting points for the corresponding simulations. Molecular dynamics simulations were performed using the GROMACS 2019.25 molecular dynamics package (Abraham et al., 2015). An AMBER99SB-ILDN (Lindorff-Larsen et al., 2010) force field was used for simulations. All ligands were parameterized using ANTECHAMBER to generate parameters that are consistent with the General Amber Force Field (GAFF) (Wang et al., 2004). The AM1-BCC method was used to assign charges. ACPYPE (da Silva and Vranken, 2012) was used to prepare the ligands’ topologies into a format compatible with GROMACS. All simulations were performed in an explicit water environment, using the TIP3P model (Mark and Nilsson, 2001). Complexes were solvated in a dodecahedron box system and were neutralized with the addition of Na^+^ and Cl^−^ ions. Steepest descent was used for minimization and Fmax was set to no greater than 1000 kJ mol^−1^ nm^−1^. Systems were equilibrated using NVT and NPT ensembles for a duration of 200 ps each. The temperature was sustained at 300 K using the V-rescale (Bussi et al., 2007) algorithm. For the regulation of system pressure, a Parrinello–Rahman barostat (Parrinello et al., 1981) was used. The LINCS (LINear Constraint Solver) algorithm was used (Hess B et al., 1997) to assess bond length constraints. The particle-mesh Ewald (PME) method (Darden T et al., 1993) was used for long-range electrostatic calculations. For all simulations, the timestep was set to 2 fs. The Van der Waals cut-off distance was set to 1 nm. Binding free energies were calculated using the g_mmpbsa program (Kumari and Kumar, 2014), with the MM-PBSA method adapted for GROMACS. Binding energy consists of three energetic terms: potential energy in a vacuum, polar-solvation energy, and non-polar solvation energy. The SASA (solvent accessible surface area) model was used to calculate the non-polar solvation energy. For the calculations of the all-interaction energies, the last 5 ns (every frame, with a 100 ps interval) of the trajectories were used.

## 3. Results and Discussion

**Figure 1:**
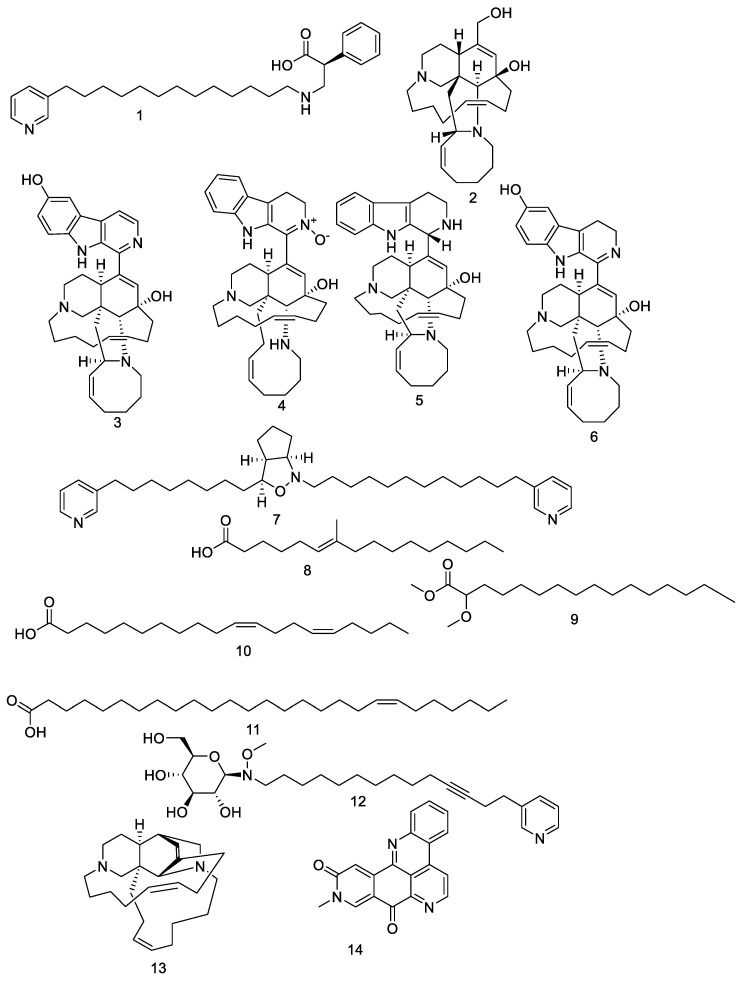
Structures of compounds **1**–**14**.

The petroleum ether fraction was first chromatographed on a silica-gel column, and was then subjected to a sephadex LH-20 column and final purification on semi-preparative HPLC, yielding compounds **1**–**14**. These 14 compounds, as shown in Figure 1, were identified based on their HR-ESIMS and by comparison with the literature, as nakinadine B (**1**), ircinol A (**2**), 6-hydroxymanzamine A (**3**), 3,4-dihydromanzamine A, J N-oxide (**4**), manzamine D (**5**), 3,4-dihydro-6-hydroxymanzamine A (**6**), pyrinodemin D (**7**), 7-methyl-6-hexadecenoic acid (**8**), methyl 2-methoxyhexadecanoate (**9**), 11,15-icosadienoicacid (**10**), 20-hepacosenoic acid (**11**), amphimedoside C (**12**), keramaphidin B (**13**), and amphimedine (**14**) [16].

### 3.1. RdRp

**Figure 2:**
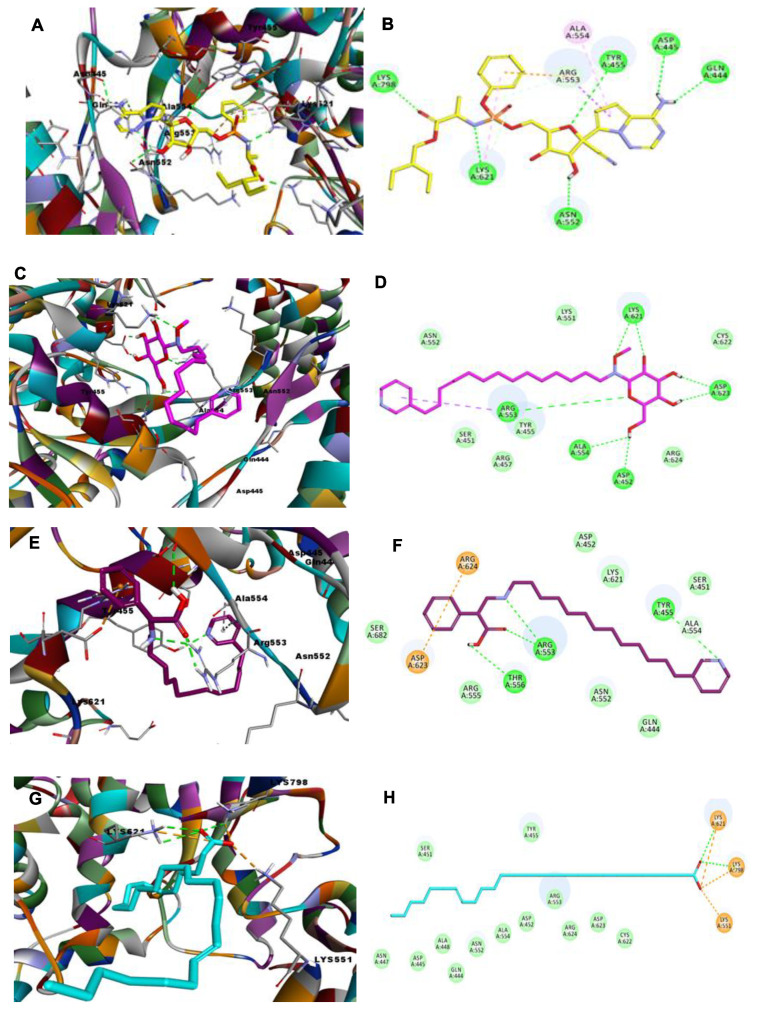
Depiction of 3D and 2D binding modes of remdesivir (**A**,**B**) and compounds **12** (**C**,**D**), **1** (**E**,**F**), and **11** (**G**,**H**) into the active site of SARS-CoV-2 RdRp (PDB entry: 6NUR).

**Figure 3:**
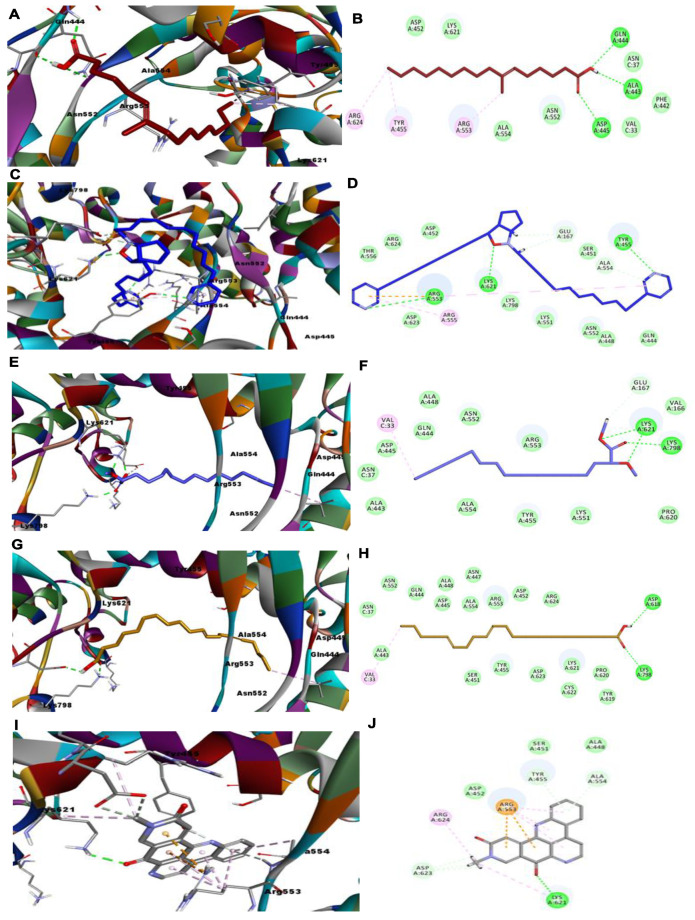
Depiction of 3D and 2D binding modes of compounds **8** (**A**,**B**), **7** (**C**,**D**), **9** (**E**,**F**), **10** (**G**,**H**), and **14** (**I**,**J**) into the active site of SARS-CoV-2 RdRp (PDB entry: 6NUR).

Docking studies were carried out for all compounds, as shown in Figure 2. Remdesivir formed six H-bonds with Gln444, Asn552, Asp445, Tyr455, Lys621, and Lys798, in addition to demonstrating hydrophobic interactions with Ala554, Lys621, and Arg553. The CDOCKER energy of remdesivir is −30.1162 and the CDOCKER interaction energy is −59.1337 (Table S1). Among the fourteen virtually screened compounds, compound **12** was the best, forming seven H-bonds with Asp623 (2), Lys621 (2), Ala554, Aps452 and Arg553, and demonstrating one hydrophobic interaction with Arg553, as shown in Figure 3. The binding sites of compound 12 were consistent with those of remdesivir; however, compound 12 showed one more extra hydrogen bond with Arg553. Notably, the CDOCKER energy (−33.0222) and the CDOCKER interaction energy (−57.2272) of compound 12 are very close to that of remdesivir, as shown in Figure 2C,D.

The data revealed that compound **1** has a CDOCKER energy of −45.4891 and a CDOCKER interaction energy of −51.245; the high CDOCKER energy draws particular attention to this compound. In addition, it is involved in the formation of four H-bonds, as is remdesivir. Compound **1** forms hydrogen bonds with the important amino acid residues Arg553 (2), Thr556, and Tyr455, and undergoes two pi-cation interactions with Arg624 and Asp623 and one hydrophobic interaction with Asp623, as illustrated in Figure 2E,F. Compound **11** (Figure 2G,H), is engaged in the formation of two hydrogen bonds with the amino acid residues Lys621 and Lys798, in addition to three attractive charge and salt bridge interactions, which may explain its lower CDOCKER energy (−40.4527) and CDOCKER interaction energy (−55.0797).

Compounds **8**, **7**, and **9** showed low binding energy, as shown in Table S1, and formed three H-bonds, as shown in Figure 3A–F. Compound **10** engaged in the formation of two H-bonds with Asp618 and Lys798 and displayed one hydrophobic interaction with Val33. Finally, compound **14** showed the lowest activity, with a CDOKER energy of −2.54925 and a CDOCKER interaction energy of −31.7949, forming one hydrogen bond with Lys621. Compounds **2**, **3**, **4**, **5**, **6,** and **13** showed positive CDOCKER energy readings, indicating that they are inactive as SARS-CoV-2 RdRp inhibitors.

**Figure 4:**
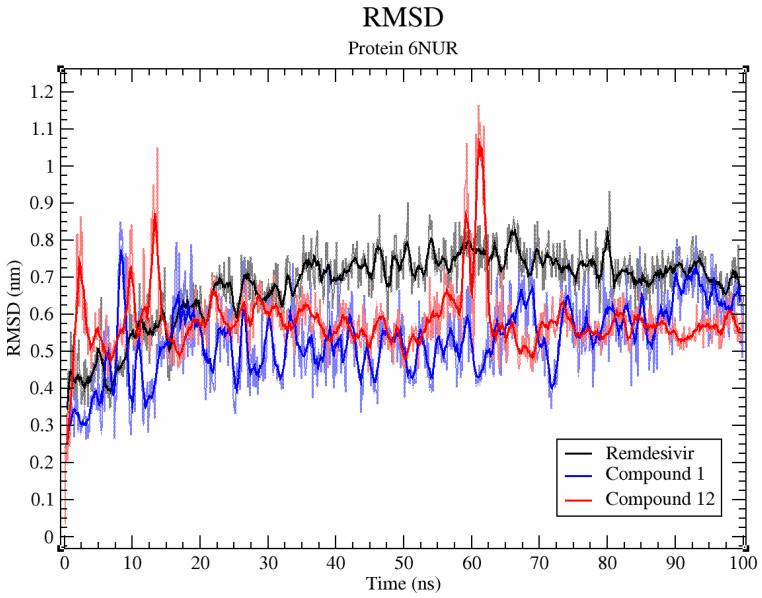
RMSD plot of compounds **1**, **12**, and remdesivir during a 100 ns MD simulation with SARS-CoV-2 RdRp.

Based on the analysis of the molecular docking results, compounds **1** and **12** were selected for molecular dynamics simulation studies. Compounds **1** and **12**, similarly to remdesivir, maintain relative stability (Figure 4, RMSD < 0.2 nm) when interacting with SARS-CoV-2 RdRp (PDB ID 6NUR).

**Figure 5:**
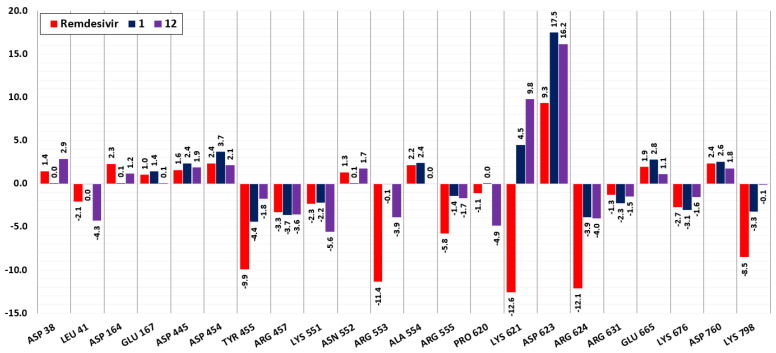
Total contribution energy (kJ/mol) of the commonly interacting amino acid residues of the RdRp protein for compounds **1**, **12** and remdesivir.

Compounds **1**, **12**, and remdesivir interact with the SARS-CoV-2 RdRp (PDB ID 6NUR) with very similar total binding energies: −45.1, −47.9, and −44.8 kJ/mol, respectively. A comparison of the total contribution energies (ΔG_total_ = ΔE_MM_ + ΔG_polar_ + ΔG_apolar_) of the common interacting amino acid residues of the RdRp protein for compounds **1**, **12**, and remdesivir is presented in Figure 5. Even though the average total binding energies for all interacting amino acid residues of RdRp with the three studied compounds are similar, among the commonly interacting residues, remdesivir presents more optimal energy values (Figure 5).

Remdesivir has exceptionally low binding energy values with the following amino acids of the RdRp protein: Tyr455, Arg553, Lys621, Arg624, and Lys798. Compounds **1** and **12**, in contrast to remdesivir, interact with Lys621 with high positive energies, which results in a repulsive effect. Both remdesivir and compound **12** interact with Asp38, Asp 164, and Asn552 with positive energies, and with Leu41, Arg553, Pro620 with negative energies, whereas compound **1** does not interact with them. Both remdesivir and compound **1** interact with Glu167 and Ala554 with positive energies, and with Lys798 with negative energies, whereas compound **12** does not interact with these residues. Compound **1** also interacts with positive total energies with the amino acid residues Glu350, Asp390, Thr556, Asp618, Glu658, Gly678, and Thr680, and interacts with negative energies with Arg392, Met542, Val557, Ala558, Cys622, and Ser682.

Compound **12** also interacts with Asn37, Lys51, and Cys622 with negative total energies and with Asp452 with positive energy. The following amino acid residues interact exclusively with remdesivir: Lys7, Lys438, Lys545, Lys783, Arg836 interact with negative total energies, and Asp161, Glu436, Ala448, Asp761, Glu811, Asp833, Asp845 interact with positive total energies that weaken the binding of the compound. As can be seen from Figure 5, among the three compounds, remdesivir has significantly stronger interactions with the common interacting amino acid residues. However, the cumulative effect of the interactions of compounds **1** and **12** with other (uncommon) residues results in these compounds having similar total binding energy values to remdesivir.

### 3.2. CL Protease

**Figure 6:**
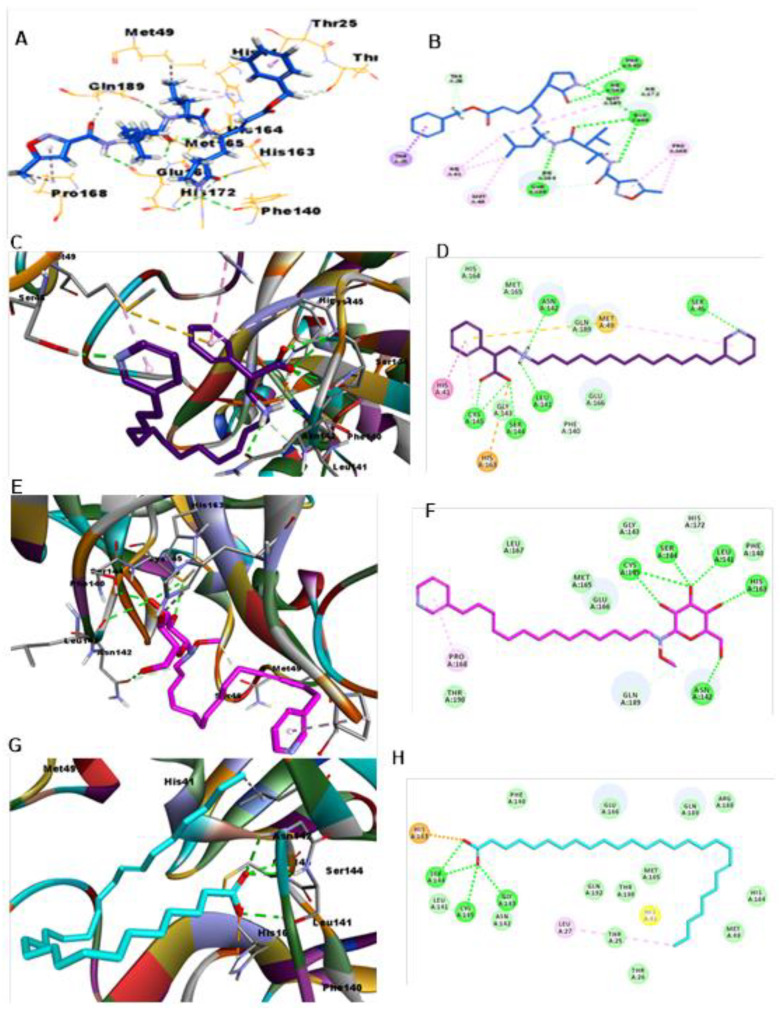
Depiction of 3D and 2D binding modes of ligand **N3** (**A**,**B**) and compounds **1** (**C**,**D**), **12** (**E**,**F**) and **11** (**G**,**H**) into the active site of 3CL protease (PDB ID: 6LU7).

Validation of the docking protocol was carried out by redocking the ligand N3 in 3CL protease (PDB ID: 6LU7). The RMSD value was less than 0.926 (less than 2), which indicates confidence in the produced docking results. The docking analysis of ligand N3 showed that it formed six H-bonds with amino acid residues Phe140, His163, Clu166 (3), and Gln189, in addition to undergoing many hydrophobic interactions with various amino acid residues, as illustrated in Figure 6**.** Interestingly, out of the fourteen virtually screened compounds, the same eight compounds which were active against the SARS-CoV-2 RdRp enzyme were also active against 3CL protease (PDB ID: 6LU7), albeit with some differences. Compound **1** was the most promising one, as it could bind well to the substrate binding pocket of 3CL protease (PDB ID: 6LU7) and showed significant inhibition and a lower docking score compared to the standard drug (Table S1). Compound **1**, surprisingly, showed the best CDOCKER energy (−56.5311) and the highest CDOCKER interaction energy (−48.047) and formed six H-bonds with amino acid residues Cys145 (2), Ser144, Leu141, Asn142, and Ser46, in addition to displaying many hydrophobic interactions with His41, Met49, Cys145, and His163 (Figure 6, Table S1). Surprisingly, compound **12**, which was the most active against the SARS-CoV-2 RdRp enzyme, also showed lower CDOCKER energy (−26.4258) and the highest CDOCKER interaction energy (−47.9309), and formed six H-bonds with Asn142, Phe140, Leu141, His163, Ser144, and Cys145, in addition to undergoing hydrophobic interactions with Asn142, His172, and Gln189, as shown in Figure 6. In compound **11,** four H-bonds were formed with residues Ser144 (2), Gly143, and Cys14, in addition to hydrophobic interactions with Leu27 and one important salt bridge with His163, which may reflect its lower CDOCKER energy (−55.0797) and CDOCKER interaction energy (−40.2121).

**Figure 7:**
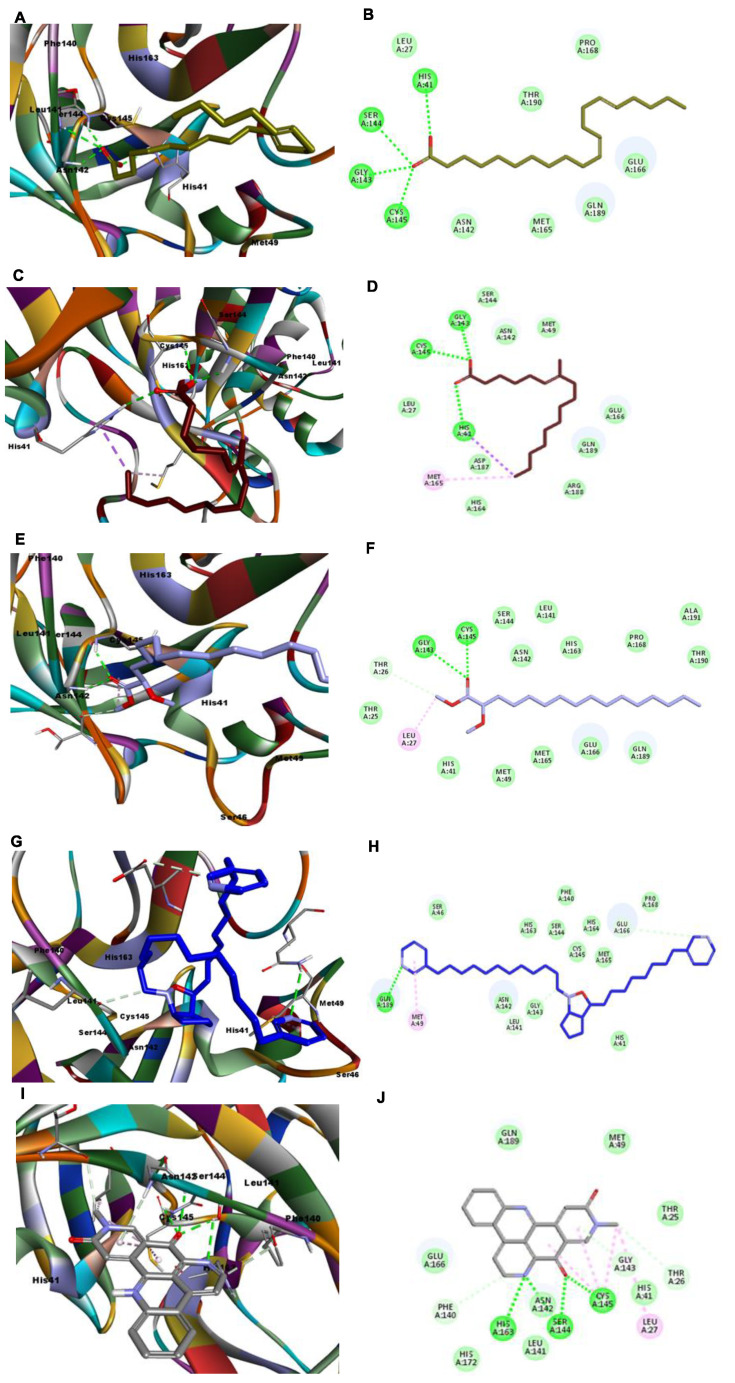
Depiction of 3D and 2D binding modes of compounds **10** (**A**,**B**), **8** (**C**,**D**), **9** (**E**,**F**), **7** (**G**,**H**), and **14** (**I**,**J**) into the active site of the 3CL protease (PDB ID: 6LU7).

In compound **10**, as shown in Figure 7A,B, four H-bonds were formed with the residues Ser144, Cys145, Gly143, and His41. Moreover, in compound **8**, three H-bonds were formed with the residues His41, Gly143, and Cys154, in addition to two hydrophobic interactions with Met49 and Met165. Notably, compound **9** formed only two H-bonds, with the residues Cys145 and Gly143, and hydrophobic interaction occurred with Leu27 and Thr26; however, it displayed a good CDOCKER energy (−39.2038) and CDOCKER interaction energy (−39.0384), as shown in Table S1 and Figure 7E,F. Compound **7** formed one H-bond with the amino acid residue Gln189 and underwent one hydrophobic interaction with the residue Met49. It displayed a CDOCKER energy of −22.9961 and a CDOCKER interaction energy of −46.8449, as shown in Table S1 and Figure 7G,H. Finally, compound **14**, which was the least active against the SARS-CoV-2 RdRp enzyme, was also the least active against 3CL protease (PDB ID: 6LU7), with a CDOCKER energy of −4.5213 and a CDOCKER interaction energy of −37.3417, as shown in Table S1 and Figure 7I,J. Again, compounds **2**, **3**, **4**, **5**, **6**, and **13** showed positive CDOCKER energy, indicating that they are inactive against 3CL protease (PDB ID: 6LU7).

The docking study revealed that the virtually screened compounds, in particular the compounds nakinadine B (**1**), 20-hepacosenoic acid (**11**), and amphimedoside C (**12**), fit well into the pockets of both enzymes and may be effective against the new coronavirus via dual inhibition of SARS-CoV-2 RdRp and 3Cl main protease.

**Figure 8:**
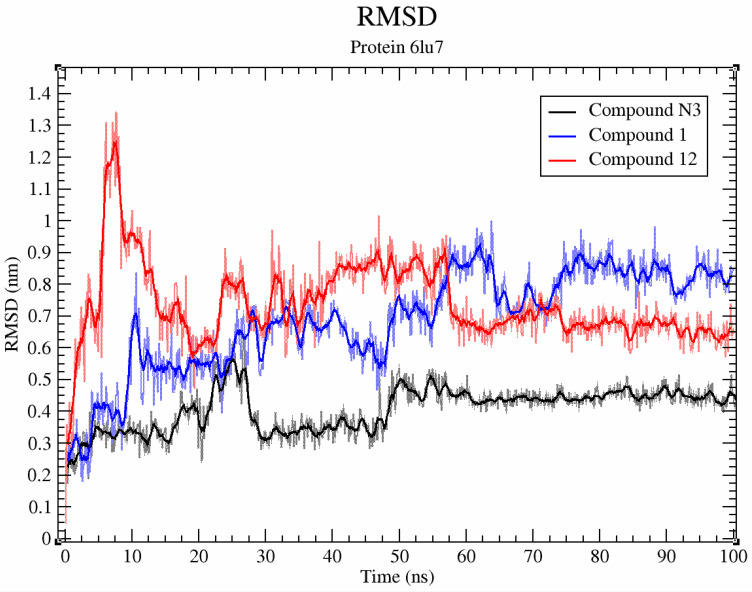
RMSD plot of compounds **1**, **12** and co-crystallized ligand (N3) during a 100 ns MD simulation with SARS-CoV-2 3CL protease.

Compounds **1** and **12**, as co-crystallized ligands (N3) (PDB ID 6LU7), are relatively stable (Figure 8, average RMSD < 0.2 nm), but undergo noticeable deviation during interaction with the SARS-CoV-2 3CL protease (PDB ID 6LU7).

**Figure 9:**
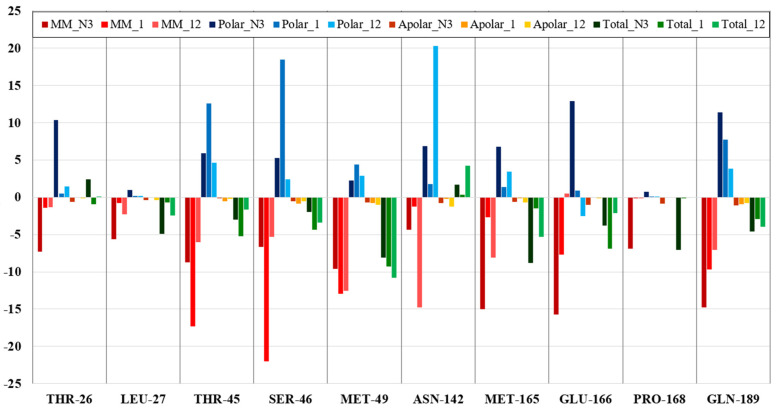
Energy contribution (kJ/mol) of the common interacting amino acid residues of the SARS-CoV-2 3CL protease for compounds **1** and **12** and co-crystallized ligand (N3).

A comparison of the total contribution energies (ΔG_total_ = ΔE_MM_ + ΔG_polar_ + ΔG_apolar_) of the interacting amino acid residues of the SARS-CoV-2 3CL protease for compounds **1** and **12** is presented in Figure 9.

Compounds **1** and **12**, in contrast to the reference ligand (N3), have higher binding affinity to Met49 and Ser46. Compound **1** has exceptionally high binding affinity towards Thr45 and Glu166. Compound **12** has a higher binding affinity to Met165 than compound **1**, but lower affinity compared to the reference ligand. Reference ligand N3 has a higher binding affinity to Leu27, Met165, and Pro168, in comparison to compounds **1** and **12**. All three compounds interact with Gln189 with relatively similar low total binding energies. Compounds **1** (nakinadine B) and **12** (amphimedoside C) both interact with the SARS-CoV-2 RdRp and 3CL protease with low binding energies. Amphimedoside C has a much lower binding energy with 3CL main protease (−127.3 kJ/mol) than nakinadine B (−69.8 kJ/mol), and is close to reference ligand N3 in this regard (−156 kJ/mol). Both nakinadine B and amphimedoside C interact with RdRp with similar low binding energies (−45.1 and −47.9 kJ/mol, respectively), values similar to the result for remdesivir (−44.8 kJ/mol). Energetic analysis of nakinadine B and amphimedoside C demonstrated that both compounds have high binding affinity for SARS-CoV-2 RdRp and 3CL main protease. The data obtained can be used for further drug design and the optimization of potential dual inhibitors of SARS-CoV-2 RdRp and 3CL main protease.

Moreover, we studied the pharmacokinetic properties of the most promising eight compounds, as shown in Table S3. The results showed that all the compounds under investigation (**1**, **7**–**12**, **14**) showed good values for oral absorption. They revealed high Caco2 permeability values (0.919, 1.127, 1.575, 1.719, 1.565, 1.264, 0.597, and 1.389, respectively) and high intestinal absorption percentages (88.201%, 90.9%, 90.63%, 92.925%, 89.723%, 86.168%, 83.364% and 100%, respectively), which reflects their potential for good oral absorption. Furthermore, all the tested compounds displayed relatively good skin permeability, with a logKp less than −2.5. One of the most significant parameters is a drug’s ability to cross into the brain, which can increase its efficacy, as its pharmacological action can occur centrally. It also presents certain advantages in studying the side effects. We observed from our results that some compounds, such as compound **12**, showed poor distribution to the brain, and some, such as compound **14**, were able to penetrate the BBB readily, whereas the remainder had a reasonable ability to penetrate the blood–brain barrier. This was based on the following protocol: molecules with an IogBB > 0.3 are considered able to readily cross the blood–brain barrier, whereas molecules with an IogBB < −1 are poorly distributed to the brain. Moreover, when CNS permeability was examined, it was found that all compounds had the ability to penetrate the CNS except for compound **14,** as compounds with an IogPS > −2 are considered able to penetrate the central nervous system (CNS), whereas those with IogPS < −3 are considered unable to do so. Furthermore, the volume of distribution (VDss) was measured for all the compounds. VDss is considered low if below 0.71 L/Kg (log VDss < −0.15) and high if above 2.81 L/Kg (log VDss > −0.45). We observed that compounds **7** and **14** had high VDss values, whereas compounds **8**, **10**, **11** and **12** had low values, and compounds **1** and **9** had moderate values. An important detoxification enzyme, chiefly found in the liver, is Cytochrome P450 [25]. Cytochrome P450 activates many drugs and deactivates others. Therefore, it is necessary to investigate the compound’s ability to inhibit this enzyme. As shown in Table S2, it was determined whether the compounds were cytochrome P450 inhibitors or not. It was noted that the parameters related to metabolism and excretion showed no remarkable differences among all the tested compounds. AMES toxicity was detected and all of the tested compounds were found not to be mutagenic and therefore not to act as carcinogenic agents. Moreover, the inhibition of hERG channels has resulted in the removal of many substances from the pharmaceutical market, so our compounds were examined in order to ascertain whether they acted as hERG I and II inhibitors. Our results revealed that none of the compounds acted as inhibitors against hERG I, and that only three of them, **7**, **10**, and **12,** inhibited hERG II. Furthermore, a hepatotoxicity prediction was carried out on our tested compounds; most showed no hepatotoxic effects, with the exception of compounds **1**, **12**, and **14**. Finally, skin sensitization is a possible side effect of dermally applied products, so our compounds were tested for their skin sensitization effects. Most compounds did not show any skin sensitization effect, with the exception of compounds **8**–**11**.

## 4. Conclusions

The docking study of fourteen compounds from *Amphimedon* sp. showed that the virtually screened compounds might be effective against the new coronavirus via the dual inhibition of SARS-CoV-2 RdRp and 3CL main protease. In particular, the compounds nakinadine B (**1**), 20-hepacosenoic acid (**11**), and amphimedoside C (**12**) were the most promising, as they were well accommodated in the pockets of both enzymes. Moreover, the molecular dynamic study revealed that compounds **1** (nakinadine B) and **12** (amphimedoside C) both interacted with the SARS-CoV-2 RdRp and 3CL protease with low binding energies. Amphimedoside C had the lowest binding energy with 3CL main protease (−127.3 kJ/mol), a value which is double that of nakinadine B (−69.8 kJ/mol). Both nakinadine B and amphimedoside C interact with RdRp with similarly low binding energies (−45.1 and −47.9 kJ/mol, respectively). Additionally, the pharmacokinetic properties of the most promising eight compounds (**1**, **7**–**12**, **14**) showed that they all had good oral absorption values. They also revealed high Caco2 permeability (0.919, 1.127, 1.575, 1.719, 1.565, 1.264, 0.597 and 1.389, respectively) and high intestinal absorption (88.201%, 90.9%, 90.63%, 92.925%, 89.723%, 86.168%, 83.364% and 100%, respectively), which reflects a good potential for oral absorption with limited toxicity. Taken together, the data from this screening study indicate the importance of *Amphimedon* sp. as a potential source of multitargets acting on COVID-19, and highlight its potential in the rapid discovery of drugs from natural sources.

## Data Availability

Not applicable.

## Supplementary Materials

The following are available online. Table S1: CDOCKER energy and CDOCKER interaction energy of the virtually screened compounds, Remdesivir and Ligand N3 on RdRp and 3CL pro enzymes, Table S2: Different interaction energy types and total binding energies of compounds **1** and **12** with SARSCoV-2 RdRp (PDB ID 6NUR) and 3CL protease (PDB ID 6LU7), Table S3: Pharmacokinetic properties of compounds.
