# Supplementary material for: Targeting 3CLpro and SARS-CoV-2 RdRp by Amphimedon sp. Metabolites: A Computational Study
Source: Molecules. 2021 Jun 21;26(12):3775. doi: 10.3390/molecules26123775 (PMC8235472; doi:10.3390/molecules26123775)
Supplement: Supplementary file 1 [file molecules-26-03775-s001.zip › molecules-1250751-supplementary.pdf]

Targeting 3CLpro and SARS-CoV-2 RdRp by *Amphimedon* sp. metabolites: a computational study

Nourhan Hisham Shady [a], Alaa M. Hayallah [b,c], Mamdouh F. A. Mohamed [d], Mohammed M. Ghoneim [e,f], Garri Chilingaryan[g], Mohammad M. Al-Sanea [h], Mostafa A. Fouad [i] , Mohamed Salah Kamel [a,i] and Usama Ramadan Abdelmohsen [a,i]*

[a] Department of Pharmacognosy, Faculty of Pharmacy, Deraya University, Universities Zone, P.O. Box 61111 New Minia City, Minia, Egypt.

[b] Department of Pharmaceutical Organic Chemistry, Faculty of Pharmacy, Assiut University, Assiut 71526, Egypt.

[c] Pharmaceutical Chemistry Dept. Faculty of pharmacy, Sphinx University. New Assiut. Egypt.

[d] Department of Pharmaceutical Chemistry, Faculty of Pharmacy, Sohag University, 82524 Sohag, Egypt.

[e] Department of Pharmacy Practice,College of Pharmacy, AlMaarefa University, Ad Diriyah 13713, Saudi Arabia.

[f] Department of Pharmacognosy, Faculty of Pharmacy, Al-Azhar University, Cairo, 11371, Egypt

[g] Institute of Biomedicine and Pharmacy, Russian –Armenian University, Armenia.

[h] Department of Pharmaceutical Chemistry, College of Pharmacy, Jouf University, Aljouf 72341, Saudi Arabia.

[i] Department of Pharmacognosy, Faculty of Pharmacy, Minia University, 61519 Minia, Egypt

* Correspondence: usama.ramadan@ mu.edu.eg, Tel.: +2-86-2347759, Fax: +2-86-2369075 (URA);

Table S1. The CDOCKER energy and CDOCKER interaction energy of the virtually screened compounds, Remdesivir and Ligand N3 on RdRp and 3CL pro enzymes.

	RdRp (PDB ID: 6NUR)		3CL protease (PDB ID: 6LU7).	
**Compound**	**CDOCKER energy**	**CDOCKER interaction energy**	**CDOCKER energy**	**CDOCKER interaction energy**
1	-45.4891	-51.245	-56.5311	-48.047
7	-24.9113	-55.4676	-22.9961	-46.8449
8	-14.9527	-33.013	-18.0475	-40.973
9	-34.6093	-36.2722	-39.2038	-39.0384
10	-15.8932	-43.8066	-16.4872	-50.1956
11	-40.4527	-55.0797	-40.2121	-50.9693
12	-33.0222	-57.2272	-26.4258	-47.9309
14	-2.54925	-31.7949	-4.5213	-37.3417
Remdesivir	-30.1162	-59.1337	ND	ND
Ligand N3	ND	ND	-70.8463	-79.0435

Table S2. Different interaction energy types and total binding energies of compounds 1 and 12 with SARS-CoV-2 RdRp (PDB ID 6NUR) and 3CL protease (PDB ID 6LU7).

	**Van der Waals (kJ/mol)**	**Electrostatic (kJ/mol)**	**Polar Solvation (kJ/mol)**	**SASA (kJ/mol)**	**Binding Energy (kJ/mol)**	**Standard deviation (kJ/mol)**
**RdRp**						
Remdesivir	-135.85	-94.41	202.29	-16.83	-44.77	± 2.62
Compound 1	-203.89	-32.99	215.63	-23.69	-45.12	± 2.45
Compound 12	-148.28	-135.13	255.7	-20.4	-47.86	± 2.33
**3CL protease**						
Compound N3	-284.57	-96.25	251.63	-26.83	-156.02	± 2.46
Compound 1	-149.93	-87.39	183.66	-16.02	-69.81	± 2.03
Compound 12	-198.6	-33.42	123.96	-19.02	-127.33	± 1.79

Table S3. Pharmacokinetic properties of compounds.

Property		Compound 1	Compound 7	Compound 8	Compound 9	Compound 10	Compound 11
Absorption	Water Solubility (log mol/L)	-2.94	-6.781	-6.419	-6.309	-7.183	**-5.578**
	Caco2 permeability (log Papp in 10^{-6} cm/s)	0.919	1.127	1.575	1.719	1.565	**1.264**
	Intestinal absorption (%)	88.201	90.9	90.63	92.925	89.723	**86.168**
	Skin permeability (log Kp)	-2.73	-2.758	-2.661	-2.681	-2.758	**-2.734**
	P-glycoprotein substrate	Yes	No	No	No	No	**Yes**
	P-glycoprotein I	No	Yes	No	No	No	**No**
	P-glycoprotein II	Yes	Yes	No	No	Yes	**Yes**
Distribution	VDss (log L/kg)	-0.113	0.566	-0.255	0.237	-0.295	-0.644
	Fraction unbound (Fu)	0.349	0	0.085	0.116	0.034	**0.098**
	BBB permeability (log BB)	-0.477	-0.396	-0.243	0.224	-0.393	**-0.731**
	CNS permeability (log PS)	-2.709	-2.118	-1.831	-1.984	-1.507	**-0.559**
Metabolism	CYP2D6 substrate	Yes	No	No	No	No	No
	CYP3A4 substrate	Yes	Yes	Yes	Yes	Yes	Yes
	CYP1A2 inhibitor	No	No	Yes	Yes	Yes	No
	CYP2C19 inhibitor	No	No	No	No	No	No
	CYP2C9 inhibitor	No	No	No	No	No	No
	CYP2D6 inhibitor	No	No	No	No	No	No
	CYP3A4 inhibitor	No	No	No	No	No	No
Excretion	Total Clearance (log ml/mim/kg)	1.184	1.605	1.784	1.982	1.996	1.985
	Renal OCT2 substrate	No	No	No	No	No	No
Toxicity	AMES toxicity	No	No	No	No	No	No
	Max. tolerated dose (log mg/kg/day)	-0.512	0.238	-0.305	0.317	-0.676	**-1.166**
	hERG I inhibitor	No	No	No	No	No	**No**
	hERG II inhibitor	No	Yes	No	No	Yes	**No**
	Oral Rat Acute Toxicity (LD50) (mol/kg)	2.719	2.041	1.754	1.67	1.749	**3.158**
	Oral Rat Chronic Toxicity (LOAEL) (mol/kg_bw/day)	1.823	2.624	3.095	2.727	3.313	**3.86**
	Hepatotoxicity	Yes	No	No	No	No	**No**
	Skin Sensitisation	No	No	Yes	Yes	Yes	**Yes**

	T.Pyriformis toxicity (log µg/L)	0.293	0.336	1.702	1.7	0.932	**0.319**
	Minnow toxicity (log mM)	**-1.235**	**-4.33**	-0.975	-1.335	**-1.607**	**-2.736**

Property		Compound 12	Compound 14			
Absorption	Water Solubility (log mol/L)	**-3.788**	**-3.09**			
	Caco2 permeability (log Papp in 10^{-6} cm/s)	**0.597**	**1.389**			
	Intestinal absorption (%)	**83.364**	**100**			
	Skin permeability (log Kp)	**-2.742**	**-2.733**			
	P-glycoprotein substrate	**Yes**	**No**			
	P-glycoprotein I	**Yes**	**No**			
	P-glycoprotein II	**No**	**Yes**			
Distribution	VDss (log L/kg)	**-1.3**	**0.458**			
	Fraction unbound (Fu)	**0.235**	**0.336**			
	BBB permeability (log BB)	**-1.608**	**0.607**			
	CNS permeability (log PS)	**-3.491**	**-2.057**			
Metabolism	CYP2D6 substrate	**No**	**No**			
	CYP3A4 substrate	**Yes**	**Yes**			
	CYP1A2 inhibitor	**No**	**Yes**			
	CYP2C19 inhibitor	**No**	**Yes**			
	CYP2C9 inhibitor	**No**	**Yes**			
	CYP2D6 inhibitor	**No**	**No**			
	CYP3A4 inhibitor	**No**	**Yes**			
Excretion	Total Clearance (log ml/mim/kg)	**1.762**	**0.835**			
	Renal OCT2 substrate	**No**	**No**			
Toxicity	AMES toxicity	**No**	**No**			
	Max. tolerated dose (log mg/kg/day)	**-0.058**	**0.425**			
	hERG I inhibitor	**No**	**No**			
	hERG II inhibitor	**Yes**	**No**			
	Oral Rat Acute Toxicity (LD50) (mol/kg)	**4.162**	**2.61**			
	Oral Rat Chronic Toxicity (LOAEL) (mol/kg_bw/day)	**4.417**	**0.649**			
	Hepatotoxicity	**Yes**	**Yes**			
	Skin Sensitisation	**No**	**No**			

	T.Pyriformis toxicity (log µg/L)	**0.285**	**0.285**			
	Minnow toxicity (log mM)	**2.319**	**0.612**			
